# Associations between socio-demographic factors and diet patterns among Indian women in a low-income urban setting

**DOI:** 10.1038/s41430-026-01739-w

**Published:** 2026-05-07

**Authors:** Sarah H. Kehoe, Harsha Chopra, Devi Shivshankaran, Chiara Di Gravio, Sirazul A. Sahariah, Meera Gandhi, Barrie M. Margetts, Ramesh D. Potdar, Caroline H. D. Fall

**Affiliations:** 1 Medical Research Council Lifecourse Epidemiology Centre, Tremona Road, Southampton, SO16 6YD UK; 2 Centre for Study of Social Change, Mumbai, India; 3 Department of Epidemiology and Biostatistics, School of Public Health, Imperial College London, London, W2 1PG UK; 4 DY Patil School of Public Health, DY Patil Deemed to be University, Nerul, Navi Mumbai, India; 5 School of Human Development and Health in Faculty of Medicine, University of Southampton, Tremona Road, Southampton, SO16 6YD UK

**Keywords:** Epidemiology, Risk factors

## Abstract

**Background/objectives:**

Improving the diets of women of reproductive age living in Mumbai slums may benefit their own health and that of their children. Our objective was to identify diet patterns in this population group.

**Subjects/methods:**

Participants were married women (*n* = 6513) aged <40 years enroled in a randomised controlled trial of a food-based intervention. We collected dietary data at the time of enrolment using a 212-item food frequency questionnaire. We used principal component analysis and cluster analysis to identify dietary patterns and explored socio-demographic correlates of the patterns using multivariate linear regression models.

**Results:**

We identified 2 interpretable patterns. The first, named the ‘Snack, Fruit and Sweet’ pattern was characterised by vegetable curry, fried snacks, salad, fruit, sweets and sweetened beverages and explained 9.3% of the variance. Scores on this pattern were independently associated with the women not working versus being employed (0.13, CI: 0.06, 0.19, *p* < 0.001) and with standard of living index score (0.03, CI: 0.02, 0.04, *p* < 0.001). The second pattern was named ‘Non-vegetarian’ and was characterised by meat and biriyani and rice and noodle dishes, with low intakes of lentils, legumes, nuts and seeds. This pattern was associated with being Muslim and was negatively associated with age (-0.02, CI: -0.03, -0.02, *p* < 0.001) and standard of living index (-0.01, CI: -0.01, 0.00, *p* < 0.001). It explained 6.7% of the variance.

**Conclusions:**

We identified two interpretable patterns that can be used to better understand dietary habits in Mumbai slums. This knowledge can be used to target interventions aimed at improving diet quality in these communities.

## Introduction

The pre-conceptional diets of women of reproductive age are important for their own health and also for the short and long term health of their children [1, 2]. The rapid urbanisation occurring in India coupled with the nutrition transition [3, 4] is leading to changes in diet and the prevalence of under- and over-nutrition in India [5].

In 2020 approximately 239 million people in India lived in slums, equivalent to 49% of the Indian urban population [6]. An estimated 60% of Mumbai inhabitants live in slum conditions, defined by the World Bank as a group of individuals living under the same roof, lacking one or more of the basic necessities (including water, sanitation, sufficient living space, housing durability, security of tenure) [7]. Infrequent consumption of micronutrient-rich foods such as fruit, vegetables, milk, meat, fish and eggs has been described in Mumbai slums [8], yet little is known about the diet patterns of women living in these communities.

The traditional approach to studying diet has been to investigate associations between single nutrients and/or foods and outcomes of interest [9, 10]. In reality, nutrients and foods are not normally consumed in isolation so determining which foods are commonly eaten together as part of a diet pattern is likely to be more representative of typical eating behaviour. Evidence suggests that interventions aimed at altering diet patterns may be more effective in terms of reducing the health risks associated with poor diet than single nutrient interventions [10]. Diet patterns have been shown to be associated with socio-demographic factors e.g. age [11–15], socio-economic status and educational attainment [16–19]. Knowledge of such associations may improve the design and targeting of interventions aimed at increasing intakes of micronutrient-rich foods.

To date, the vast majority of diet patterns research has been conducted in the USA and Europe [10]. Despite the fact that diet patterns are likely to be population-specific, there are few data from low and middle income countries (LMIC) including India [20]. In a previous exploratory study we identified two diet patterns among 9 year old children living in rural and urban areas in and around Mysore, South India [21]. These patterns described marked variability in food choice and diet quality. Furthermore, they predicted variation in blood nutrient biomarkers and were related to differences in body composition. There is evidence that dietary patterns differ widely across India [20] and there has been little study of patterns among slum-dwelling women of reproductive age. It is important to study this group as they make up a large proportion of the population and diet during preconception and pregnancy affects the mother’s health, for example her risk of gestational diabetes [2] and the lifelong health of her children [22, 23].

Our aims were to identify diet patterns among women of reproductive age in a Mumbai slum and to investigate associations between these patterns and socio-demographic factors.

## Methods

### Participants & setting

We used baseline data collected from a large population of women (*n* = 6513) enroled in a randomised controlled trial of a food-based intervention named the Mumbai Maternal Nutrition Project (MMNP) [1]. Women living in slums in the Bandra, Khar, Santa Cruz and Andheri areas of Mumbai, India were invited to participate by community health workers in their area. Women were eligible for the MMNP if aged <40 years, married, non-pregnant, not sterilised, positively planning to have children and intending to deliver in Mumbai.

### Ethical approval

The study (www.controlled-trials.com/isrctn/; ISRCTN62811278) was granted ethical approval by the committees of BYL Nair and TN Medical College, Grant Medical College, and Sir JJ Group of Hospitals, Mumbai, and Southampton and SW Local Research Ethics Committees. Informed consent was obtained from all participants and all methods were performed in accordance with the relevant guidelines and regulations.

### Procedure

Data on age, religion, education, occupation and socio-economic status were collected by interview. For socio-economic status assessment we used the Standard of Living questionnaire from the Indian National Family Health Survey [24]. Height was measured to the nearest 0.1 cm using a portable stadiometer (Microtoise, UK); weight to the nearest 100 g using electronic scales (Salter, UK). A food frequency questionnaire (FFQ) was also administered (see Dietary Assessment).

### Dietary assessment

A 212-item FFQ (OSM) was developed specifically for use in the MMNP following detailed focus group discussions with the women from the study area and consultation with local nutritionists to ensure all foods consumed by women in the community were included. The FFQ was administered in Hindi or Marathi by a nutritionist or trained project assistant at the time of enrolment to the MMNP. The reference period for the FFQ was the previous seven days and the women were asked whether their usual intake was affected by factors such as illness or festivals.

Women were asked about frequency of their intake of the 212 foods and could respond with a daily or weekly frequency e.g. ‘once per day’, ‘twice per day’ or ‘3 times per week’.

Energy intakes were calculated using McCance and Widdowson’s ‘The Composition of Foods’ integrated dataset versions 5 and 6 [25]. For foods prepared at home (*n* = 164), at least two household recipes were obtained from women living in the study area, energy content was calculated as described below and values were averaged. For each recipe, all ingredients were weighed in the project kitchen to the nearest gram using electronic scales. The food was then cooked and weighed. The energy content per 100 g of the cooked food was calculated as follows:

$$\mathrm{Energy}\,\mathrm{content}/\,100{\rm{g}}\,\mathrm{of}\,\mathrm{cooked}\,\mathrm{food}\,=\frac{\mathrm{Total}\,\mathrm{energy}\,\mathrm{content}\,\mathrm{of}\,\mathrm{raw}\,\mathrm{ingredients}\,\,{\rm{x}}\,100}{\mathrm{Weight}\,\mathrm{of}\,\mathrm{cooked}\,\mathrm{food}}$$

### Data analysis methods

#### Diet patterns analysis

Prior to the diet patterns analysis, the 212 foods on the FFQ were condensed to 31 food groups based on nutrient content and typical use. For example; samosa (deep fried pastry with a potato filling), bhajia (deep fried onion in batter) and wada (deep fried potato in batter) were grouped as ‘fried snacks’. Principal Component Analysis was used to identify the women’s diet patterns. We generated components with and without Varimax rotation. PCA produces new variables (components) that are independent linear combinations of the dietary variables accounting for maximum variance [26]. The input variables were the frequency of consumption of foods from each of the 31 groups.

To adjust for unequal variance of the original variables, PCA was based on the correlation matrix. The number of components to retain was based on the identification of the point of inflexion on the scree plot, the component eigenvalue being >1, and interpretability of the component as a plausible diet pattern for the study population. Food groups with factor loadings >|0.2| were arbitrarily considered to be discriminatory. The Kaiser-Meyer-Olkin measure of sampling adequacy was used to determine whether the sample size was sufficient relative to the number of input variables [27]. Pattern scores were calculated for each woman by multiplying the input variable (i.e. frequency of intake of a food group) by the component coefficient for that food group, and summing all of these values. To assess for robustness of PCA, the analysis was repeated on a randomly selected half of the population and the results were compared to those obtained in the full population.

We also conducted a cluster analysis to determine whether different patterns were obtained compared with PCA. The 31 foods and food groups were standardised to z-scores prior to cluster analysis. Ward’s method was used to generate clusters. The resulting tree diagram was then used to decide upon the number of clusters and K-means analysis was used to assign individual participants to a particular cluster.

### Statistical analysis

Skewed variables were normalised using Fisher-Yates transformations. Linear regression models were used to assess univariate associations between socio-demographic variables and pattern scores. All variables that were significantly associated with pattern score in the univariate analyses were entered into multivariate regression models to identify independent associations with pattern scores. All statistical analyses were performed using STATA V.14 (Stata Corporation, College Station, TX).

## Results

### Participant characteristics

The mean (SD) age of the women was 25 (4) years. Mean (SD) height was 151.3 (5.5) cm and median (IQR) BMI was 20.1 (17.9,23.0) kg/m^2^. The majority (70%) were Hindu with 26% being Muslim and the remaining 4% of other faiths. Twelve per cent of women had been educated for 5 years or less, 70% for between 5 and 10 years, and 18% for more than 10 years. Over three quarters of the women (78%) were not in paid work at the time of the study. Of those in paid work, 8% were employed in unskilled jobs, 11% in skilled work and 3% in professional positions. Only 2% of the women’s husbands were not working; of those that did work 17% were employed in unskilled jobs, 65% in skilled jobs and 16% were in professional employment (Table 1).

**Table 1:** Descriptive characteristics of women studied.

	Median	IQR
**Age** (years) (*n* = 6426)	25	22 – 28
**Body Mass Index** (kg/m^2^) (*n* = 6420)	20.0	17.9 – 22.9
	**Mean**	**SD**
**Standard of Living Index score** (*n* = 6077)	24.5	6.1
	**n**	**%**
**Age** (*n* = 6426)		
Aged 16 – 18 years	188	2.9
Aged ≥19 years	6238	97.1
**Education** (*n* = 6419)		
>12 years	1130	17.6
7-12 years	4491	70.0
<6 years	797	12.4
**Religion** (*n* = 6420)		
Hindu	4525	70.5
Muslim	1620	25.2
**First language** (*n* = 6416)		
Marathi	3314	51.7
Hindi	2408	37.5
Other	694	10.8
**Season of Recruitment** (*n* = 6425)		
Winter (January – February)	1467	22.8
Pre-monsoon (March – May)	1146	17.8
Monsoon (June – September)	2199	34.2
Post-monsoon (October – December)	1613	25.1
**BMI** (*n* = 6420)		
Underweight (BMI < 18.5 kg/m^2^)	2060	32.1
Normal Range (BMI 18.5–25 kg/m^2^)	3446	53.6
Overweight (BMI > 25 kg/m^2^)	737	11.5
Obese (BMI > 30 kg/m^2^)	177	2.8

The majority (79%) of households that the women lived in were single-room dwellings. Nearly two thirds of the women lived in joint rather than nuclear households and 39% of households comprised 5 or more members. Half of the households accessed water via a public tap and 95% of women used pit rather than flush toilets.

### Dietary intakes

Table 2 shows that there was considerable variability between women in frequency of consumption of a range of food groups. Most women consumed tea, wheat and rice on a daily basis (Table 2). Three quarters of women ate no salad in the previous week. Less than 20% of women ate fruit at least once per day and 40% of women ate GLV less than once per day. Two thirds of women did not consume chicken or meat in the previous week, half had no fish and the majority had no seafood. The most frequently consumed non-vegetarian food was eggs. Half of the women had no milk or yoghurt (except for that in tea and coffee) and 13% had milk or yoghurt every day. One third of the women had fried snacks 4 or more times per week and half had them 1-3 times. Sweet foods and biscuits were consumed at least once per week by approximately one third of women.

**Table 2:** Percentage of women consuming each food group by frequency (*n* = 6513).

	Frequency of consumption per week			
	0	1 – 3	4 – 6	≥ 7
Wheat breads	3.13	4.53	2.36	90.0
Jowar (Sorghum) breads	72.3	22.2	1.11	4.39
Plain rice	0.68	1.00	0.31	98.1
Rice or noodle dishes with vegetables	67.2	31.7	0.98	0.20
Rice dishes with lentils	60.2	337.6	1.12	1.04
Lentil and vegetable curries	1.60	14.8	23.6	60.0
Lentil curries	97.3	1.37	0.32	1.01
Legume and vegetable curries	32.4	51.9	13.4	2.24
Boiled potato	53.0	45.6	0.94	0.45
Vegetable curry without potato	47.6	49.5	2.53	0.41
Vegetable curry with potato	14.6	60.7	20.4	4.27
Green leafy vegetables	9.67	22.3	8.89	59.2
Nuts and seeds	41.1	15.7	4.22	39.0
Condiments & chutneys	34.0	49.5	7.25	9.27
Salad	76.4	21.8	0.91	0.91
Raita (yoghurt with raw vegetables)	96.9	2.98	0.08	0.02
Meat (beef, mutton, offal)	63.6	27.4	5.39	3.56
Chicken	66.1	32.1	1.64	0.18
Eggs	48.3	46.6	4.21	0.89
Fish	55.3	40.1	3.90	0.77
Seafood	94.0	5.94	0.03	0.00
Rice and meat dishes (biriyani)	85.8	13.4	0.75	0.09
Fruit	15.1	47.9	19.1	17.8
Milk and yoghurt (except tea/coffee)	49.1	33.2	3.81	13.9
Fried Savoury Snacks	20.8	52.9	17.9	9.23
Sweets, chocolate, ice cream	34.7	51.6	10.0	3.75
Biscuits	33.7	35.7	10.8	19.7
Sweetened beverages	65.7	32.0	1.54	0.75
Tea with milk	10.0	1.60	0.28	88.1
Coffee with milk	93.0	6.02	0.14	0.86
Tea without milk	94.1	0.69	0.09	5.16

### Diet patterns

Based on the criteria for defining components as meaningful patterns, two components were retained (see Figure OSM1 for scree plot). Table OSM1 shows the coefficients for each of the retained components. The KMO measure of sampling adequacy indicated that the sample size was adequate for PCA.

The first two components explained 9.3% and 6.7% of the variance respectively. For the first component, the coefficients were of greatest magnitude for vegetable dishes, condiments and chutneys, salad, fruit, fried savoury snacks, sweets and sweetened beverages. The second component had large positive coefficients for meat and biriyani (rice and meat dish) and rice and noodle dishes, and coefficients for lentil and legume curry and nuts and seeds were negative. Based on these discriminatory foods, the patterns were named ‘Snack, Fruit and Sweet’ and ‘Non-vegetarian’.

### Diet patterns and socio-demographic variables

#### Snack, Fruit and Sweet pattern

Table 3 shows that our univariate analysis found that Snack, Fruit and Sweet pattern scores were positively associated with the woman’s age, husband’s occupation skill level and standard of living scores. Women who were working had higher scores on this pattern while those who were educated to graduate level had lower scores. All of these results were significant at the *p* < 0.001 level

**Table 3:** Univariate analysis of socio-demographic variables and diet pattern scores.

	Snack, Fruit and Sweet Pattern (SD)			Non-vegetarian Pattern (SD)		
	Estimate	95% CI	*P*	Estimate	95% CI	*P*
**Age**	0.01	(0.01, 0.02)	**<0.001**	-0.04	(-0.05, -0.03)	**<0.001**
**Religion**						
Hindu	(ref.)			(ref.)		
Muslim	-0.03	(-0.08, 0.03)	0.34	1.38	(1.33, 1.42)	**<0.001**
**Education**						
Primary or less	0.20	(0.14, 0.27)	**<0.001**	-0.20	(-0.26, -0.13)	**<0.001**
Secondary	(ref.)			(ref.)		
Graduate	-0.37	(-0.45, -0.29)	**<0.001**	0.37	(0.28, 0.45)	**<0.001**
**Woman’s Occupation**						
Not working	(ref.)			(ref.)		
Working	0.15	(0.09, 0.21)	**<0.001**	-0.21	(-0.27, -0.15)	**<0.001**
**Husband’s Occupation**						
Not working	-0.04	(-0.25, 0.16)	0.67	-0.04	(-0.24, 0.16)	0.68
Semi-skilled/Unskilled	(ref.)			(ref.)		
Skilled/Self-employed	0.07	(0.02, 0.13)	**0.01**	0.29	(0.24, 0.35)	**<0.001**
Professional	0.28	(0.18, 0.38)	**<0.001**	-0.22	(-0.32, -0.12)	**<0.001**
**Standard of Living Index score**	0.03	(0.03, 0.04)	**<0.001**	-0.03	(-0.03, -0.02)	**<0.001**

Table 4 presents the multivariate model, the negative association with woman’s education remained as did the association with woman’s occupation and standard of living (Table 4). All of these results were significant at the *p* < 0.001 level. Muslim women were more likely to have higher scores on this pattern but the association was more modest. Age and husband’s occupation were not associated with this pattern.

**Table 4:** Multivariate analysis of socio-demographic variables and diet pattern scores.

	Snack, Fruit and Sweet Pattern (SD)			Non-vegetarian Pattern (SD)		
	Estimate	95% CI	*P*	Estimate	95% CI	*P*
**Age**	0.01	(0.00, 0.01)	0.07	-0.02	(-0.03, -0.02)	**<0.001**
**Religion**						
Hindu	(ref.)			(ref.)		
Muslim	0.08	(0.02, 0.14)	**0.01**	1.31	(1.26, 1.35)	**<0.001**
**Education**						
Primary or less	0.07	(-0.01, 0.13)	0.07	-0.01	(-0.07, 0.05)	0.71
Secondary	(ref.)			(ref.)		
Graduate	-0.20	(-0.29, -0.11)	**<0.001**	0.20	(0.12, 0.27)	**<0.001**
**Woman’s Occupation**						
Not working	(ref.)			(ref.)		
Working	0.13	(0.06, 0.19)	**<0.001**	-0.03	(-0.08, 0.02)	0.25
**Husband’s Occupation**						
Not working	0.00	(-0.21, 0.21)	0.99	0.04	(-0.13, 0.21)	0.64
Semi-skilled/Unskilled	(ref.)			(ref.)		
Skilled/Self-employed	0.03	(-0.18, 0.25)	0.76	0.14	(-0.03, 0.31)	0.01
Professional	0.07	(-0.16, 0.30)	0.57	0.07	(-0.12, 0.26)	0.46
**Standard of Living Index score**	0.03	(0.02, 0.04)	**<0.001**	-0.01	(-0.01, 0.00)	**<0.001**

Models were mutually adjusted.

#### Non-vegetarian pattern

Table 3 shows that in univariate models, woman’s age, husband’s occupation skill level and standard of living score were all negatively associated with Non-vegetarian pattern scores. Women who worked had lower scores on this pattern. Religion was strongly associated with the Non-vegetarian pattern with Muslim women having higher scores. Women who were more highly educated had higher scores on this pattern *p* < 0.001 for all associations.

As shown in Table 4, the multivariate analysis indicated that Muslims, younger women, graduates, women without jobs and those of lower socio-economic status were more likely to adhere to the Non-vegetarian pattern *p* < 0.001 for all associations.

### Rotation of principal components

We ran our analysis with and without Varimax rotation and found no difference in the associations with socio-demographic factors (data relating to Varimax rotation not shown). We considered the unrotated components to represent more meaningful patterns than the rotated components, therefore we chose to present the data related to the unrotated components.

### Robustness of principal component analysis

To test the robustness of the patterns obtained we repeated the analysis on a randomly selected half of the population and found that the results were comparable to those from the whole population.

Cluster analysis was also conducted to examine any differences in patterns obtained using this method (data not shown). The first cluster (*n* = 4770) was characterised by low consumption frequency for most food groups. The second cluster (*n* = 1743) was characterised by high consumption of unsweetened wheat foods, fried snacks, fruits and milk. We interpreted the findings from the cluster analysis as follows: Clusters 1 and 2 appeared to represent low and high scorers on the first principal component. The women in the first cluster consumed the foods characterised by the PCA Fruit, Snack and Sweet pattern infrequently. These women had lower calorie intakes than women in the second cluster who had high scores on the Fruit, Snack and Sweet pattern. Women in cluster 2 scored higher on PCA pattern 1 (median: 1.04; IQR: 0.68, 1.59) and lower on PCA pattern 2 (median: 0.26; IQR:-0.64, 0.90). Women in cluster 1 scored lower on both PCA pattern 1 (median: -0.46, IQR: -0.84, -0.09) and 2 (median: 0.05; IQR: -0.46, 0.56). Women in cluster 2 had higher weekly energy intakes (mean: 12,779; SD: 3661) than women in cluster 1 (mean: 8,507; SD: 2474). The difference was statistically significant (*p* < 0.001). Prevalence of low BMI ( < 18.5 kg/m^2^) was similar in the two groups (cluster1: 32%, cluster 2: 31%).

## Discussion

We collected food frequency data from a large sample of women of reproductive age living in an urban slum setting in Mumbai. We found large variability in consumption of different food groups, but that overall these women had infrequent intakes of micronutrient-rich foods such as salad, fruit, milk, meat and fish. We used principal component analysis to identify diet patterns adhered to by these women. The pattern explaining most of the variance was named the ‘Snack, Fruit and Sweet pattern’, and was largely characterised by foods that could be consumed with little preparation within the home. It may therefore represent a more modern or convenient mode of eating. The Snack, Fruit and Sweet pattern was negatively associated with education but positively with socio-economic status. Women who were working were more likely to adhere to this pattern than those who were not.

The second pattern was identified as ‘Non-vegetarian’ as it was characterised by frequent consumption of mutton, chicken, eggs and biriyani. This pattern was associated with being Muslim, and was also independently associated with younger age and lower socio-economic status. Women who were not working were more likely to adhere to this pattern. These findings may be a reflection of a departure from traditional vegetarianism among younger women of lower socio-economic status.

The patterns found here are similar to patterns identified among 9 year old children in a more middle class setting in Mysore, South India [21]. It is important to consider how these patterns, which are comprised of some beneficial elements and some high fat, sugar and salt foods (HFSS) can be used to inform food-based guidelines and recommendations aimed at this population [28].

There are few data on diet patterns from South Asia. A study in urban-dwelling Pakistani men (*n* = 355) and women (*n* = 517) of low socio-economic status identified three diet patterns [17]. Firstly, the ‘prudent’ pattern was characterised by high intakes of eggs, fish, raw vegetables, fruit juice, bananas and ‘other’ fruit. This pattern was negatively associated with plasma homocysteine concentrations and was positively associated with educational attainment. The second pattern was a ‘high animal protein’ pattern with high coefficients for meat, chicken and tea with milk and was positively associated with plasma homocysteine concentrations and waist hip ratio. Adherence to this pattern was positively correlated with education and income. The third pattern was a ‘high plant protein’ pattern which was characterised by high intakes of cooked vegetables and legumes and low intakes of meat. The high plant protein pattern was negatively associated with homocysteine concentrations.

A cross-sectional study in Bengali women living in the city of Kolkata aged 35 years and above (*n* = 701) investigated associations between cardiovascular risk factors and diet patterns [29]. Three patterns were identified. The ‘vegetable, fruit and pulses’ pattern was characterised by frequent intakes of GLV, sweets, fruit, pulses (lentils), nuts, poultry and eggs. Scores on this pattern were negatively associated with serum total cholesterol and non-high-density lipoprotein (HDL) cholesterol concentrations and adherence was associated with younger age, higher educational attainment and greater income. The second pattern was named the ‘hydrogenated and saturated fat and vegetable oil’ pattern and was characterised by frequent intakes of butter, hydrogenated oil, ghee, vegetable oils, sweets, fish, high-fat dairy foods and refined cereals. This pattern was positively associated with BMI, waist circumference and HDL cholesterol concentrations. It was also associated with younger age, higher educational attainment and greater income. The third pattern was named the ‘red meat and high-fat dairy’ pattern and was characterised by frequent intakes of red meat, high-fat dairy foods, whole grain cereals, high energy drinks and low intakes of fish, refined cereals and low fat dairy foods. Adherence to this pattern was not associated with any of the CVD risk factors measured and it was negatively associated with educational attainment and income.

Venkaiah et al. [30] performed a factor analysis with diet data from 2864 men and 3525 women living in a rural area of the state of Orissa in the Northeast of India. Over half (55%) of the women were chronically under-nourished (BMI < 18.5 kg/m^2^). The first pattern was characterised by high intakes of ‘income elastic’ foods such as milk and sugar, the second by plant foods, the third by pulses, rice and legumes and labelled ‘traditional’, the fourth by micronutrient-rich foods, GLV and fruit, the fifth by fish and seafood and the sixth by nuts, seeds and meat. Three quarters of women consumed less than 16 g of fruit per day on average. The distributions of meat and fish intakes were highly positively skewed with a very small number of women consuming any of these foods. The main contributors to their diets were cereals, millet and vegetables other than GLV.

There are several similarities and differences in these observed patterns in South Asia. Animal source foods tend to be discriminatory. Apart from the Pakistani study there do not seem to be clear ‘prudent’ patterns that fit with the Indian dietary guidelines [31]. This is of interest as similar analyses of data in Western countries have found prudent or healthy diet patterns [10]. It is possible that messages about healthy eating have been less widely distributed in India than in high income countries and therefore the concept of a healthy diet is less well recognised leading to different types of diet patterns.

A large cross-sectional survey in rural parts of India studying intakes of single food groups as opposed to dietary patterns showed that low fruit and vegetable intake was associated with low socio-economic status [32]. Maternal education was positively associated with adherence to a healthy diet pattern in Australia and the UK [18, 33].

A recent study assessing the effect of rural to urban migration in India found that urban dwelling adults consumed up to 80% more fruit than their rural counterparts [34]. It is thought this is due to wider availability of produce and greater purchasing power among urban dwellers. It may also be due to a lack of indigenous fruit growing in rural areas due to deforestation. Urban dwellers also tended to have a higher energy and fat intake. This finding fits with the Snack, Fruit and Sweet pattern that is described here.

In a country as large and diverse as India it is challenging to produce one set of food-based guidelines that will be applicable to, and can be implemented by, all. The current dual burden of under-nutrition and chronic disease [35] and the predicted shift towards higher burden of chronic disease by 2050 [36] means that it is very important to disseminate clear, targeted messages that will serve the health requirements of the population. For example, targets for fruit and vegetable consumption and recommendations to limit intakes of foods high in salt, fat and sugar.

### Strengths and limitations

We studied a large group of women in a setting where little is known about dietary patterns. The fact that the women had consented to take part in a trial means that they may not be representative of the slum population in Mumbai. We do not have dietary data from women who did not take part in the trial to investigate this. We used a detailed and comprehensive FFQ that was specifically developed for this population to identify diet patterns. We did not collect blood samples at the beginning of the trial as it was expected that this would deter women from taking part. Therefore, we could not study relationships between diet and micronutrient status which would be a recommendation for future research.

## Conclusion

The patterns identified in a cohort of female slum dwellers were associated with socio-demographic factors. There was some variability between the patterns observed in the present study and those in other settings in India. However, fruit and vegetable intake consistently appears to be positively associated with standard of living or socioeconomic status. Given the predicted increase in prevalence of obesity and chronic disease in India and other LMICs, future research should investigate how diet patterns change with the nutrition transition and how these patterns are associated with risk of disease.

## Data Availability

Data can be accessed by contacting the corresponding author.

## References

[1] Potdar RD, Sahariah SA, Gandhi M, Kehoe SH, Brown N, Sane H, et al. Improving women’s diet quality preconceptionally and during gestation: effects on birth weight and prevalence of low birth weight-a randomized controlled efficacy trial in India (Mumbai Maternal Nutrition Project). Am J Clin Nutr. 2014;100:1257–68. 25332324 10.3945/ajcn.114.084921 PMC4196482

[2] Sahariah SA, Potdar RD, Gandhi M, Kehoe SH, Brown N, Sane H, et al. A Daily Snack Containing Leafy Green Vegetables, Fruit, and Milk before and during Pregnancy Prevents Gestational Diabetes in a Randomized, Controlled Trial in Mumbai, India. J Nutr. 2016;146:1453s–60s. 27281802 10.3945/jn.115.223461 PMC4926846

[3] Popkin BM. An overview on the nutrition transition and its health implications: the Bellagio meeting. Public Health Nutr. 2002;5:93–103. 12027297 10.1079/phn2001280

[4] Popkin BM, Adair LS, Ng SW. Global nutrition transition and the pandemic of obesity in developing countries. NutrRev. 2012;70:3–21. 10.1111/j.1753-4887.2011.00456.x PMC3257829 22221213

[5] Subramanian SV, Smith GD. Patterns, distribution, and determinants of under- and overnutrition: a population-based study of women in India. Am J Clin Nutr. 2006;84:633–40. 16960179 10.1093/ajcn/84.3.633

[6] Our World In Data. Number of people living in urban slum households, 2020 2024 [Available from: https://ourworldindata.org/grapher/urban-pop-in-out-of-slums.

[7] World Bank. Metadata Glossary 2024 [updated 1/7/2024. Available from: https://databank.worldbank.org/metadataglossary/world-development-indicators/series/EN.POP.SLUM.UR.ZS#:~:text=A%20slum%20household%20is%20defined%20as%20a%20group,adopted%20in%20the%20Millennium%20Development%20Goal%20Target%207.D.

[8] Chopra H, Chheda P, Kehoe S, Taskar V, Brown N, Shivashankaran D, et al. Dietary Habits of Female Urban Slum-dwellers in Mumbai. Indian J Matern Child Health. 2012;14. PMC3568157 23400755

[9] Hu FB. Dietary pattern analysis: a new direction in nutritional epidemiology. Curr Opin Lipidol. 2002;13:3–9. 11790957 10.1097/00041433-200202000-00002

[10] Newby PK, Tucker KL. Empirically derived eating patterns using factor or cluster analysis: a review. NutrRev. 2004;62:177–203. 10.1301/nr.2004.may.177-203 15212319

[11] Costacou T, Bamia C, Ferrari P, Riboli E, Trichopoulos D, Trichopoulou A. Tracing the Mediterranean diet through principal components and cluster analyses in the Greek population. Eur J Clin Nutr. 2003;57:1378–85. 14576750 10.1038/sj.ejcn.1601699

[12] Iizumi H, Amemiya T. Eleven-year follow-up of changes in individuals’ food consumption patterns. Int J Vitam Nutr Res. 1986;56:399–409. 3804619

[13] Barker ME, McClean SI, Thompson KA, Reid NG. Dietary behaviours and sociocultural demographics in Northern Ireland. Br J Nutr. 1990;64:319–29. 2223737 10.1079/bjn19900034

[14] Ahmed F, Khan MR, Akhtaruzzaman M, Karim R, Williams G, Torlesse H, et al. Long-Term Intermittent Multiple Micronutrient Supplementation Enhances Hemoglobin and Micronutrient Status More Than Iron + Folic Acid Supplementation in Bangladeshi Rural Adolescent Girls with Nutritional Anemia. J Nutr. 2010;140:1879–86. 20702745 10.3945/jn.109.119123

[15] Correa Leite ML, Nicolosi A, Cristina S, Hauser WA, Pugliese P, Nappi G. Dietary and nutritional patterns in an elderly rural population in Northern and Southern Italy: (II). Nutritional profiles associated with food behaviours. Eur J Clin Nutr. 2003;57:1522–9. 14647216 10.1038/sj.ejcn.1601720

[16] Robinson SM, Crozier SR, Borland SE, Hammond J, Barker DJ, Inskip HM. Impact of educational attainment on the quality of young women’s diets. Eur J Clin Nutr. 2004;58:1174–80. 15054431 10.1038/sj.ejcn.1601946

[17] Yakub M, Iqbal MP, Iqbal R. Dietary patterns are associated with hyperhomocysteinemia in an urban Pakistani population. J Nutr. 2010;140:1261–6. 20463142 10.3945/jn.109.120477

[18] Ambrosini GL, Huang RC, Mori TA, Hands BP, O’Sullivan TA, de Klerk NH, et al. Dietary patterns and markers for the metabolic syndrome in Australian adolescents. Nutr Metab Cardiovasc Dis. 2010;20:274–83. 19748245 10.1016/j.numecd.2009.03.024

[19] Craig LC, McNeill G, Macdiarmid JI, Masson LF, Holmes BA. Dietary patterns of school-age children in Scotland: association with socio-economic indicators, physical activity and obesity. Br J Nutr. 2010;103:319–34. 19835641 10.1017/S0007114509991942

[20] Green R, Milner J, Joy EJ, Agrawal S, Dangour AD. Dietary patterns in India: a systematic review. Br J Nutr. 2016;116:142–8. 27146890 10.1017/S0007114516001598 PMC4890343

[21] Kehoe SH, Krishnaveni GV, Veena SR, Guntupalli A, Margetts B, Fall CHD, et al. Diet patterns are associated with demographic factors and nutritional status in South Indian children. Matern Child Nutr. 2014;10:145–58. 23819872 10.1111/mcn.12046 PMC3920637

[22] Fall C. Maternal nutrition: effects on health in the next generation. Indian J Med Res. 2009;130:593–9. 20090113

[23] The 1000 Days partnership. First thousand days 2024 [Available from: http://www.thousanddays.org/.

[24] International Institute for Population Sciences. National Family Health Survey (NFHS-2) 1998-1999. Mumbai/ Washington DC; 2000.

[25] The Food Standards Agency. McCance & Widdowson’s The Composition of Foods Integrated Dataset. Version 6, Sixth summary edition. Cambridge: The Stationary Office; 2002.

[26] Joliffe IT, Morgan BJT. Principal component analysis and exploratory factor analysis. Stat Methods Med Res. 1992;1:69–95. 1341653 10.1177/096228029200100105

[27] Hutcheson G, Sofroniou N The multivariate social scientist. London: Sage; 1999 1999.

[28] Astrup A, Dyerberg J, Selleck M, Stender S. Nutrition transition and its relationship to the development of obesity and related chronic diseases. Obes Rev. 2008;9:48–52. 18307699 10.1111/j.1467-789X.2007.00438.x

[29] Ganguli D, Das N, Saha I, Biswas P, Datta S, Mukhopadhyay B, et al. Major dietary patterns and their associations with cardiovascular risk factors among women in West Bengal, India. Br J Nutr. 2011;105:1520–9. 21272403 10.1017/S0007114510005131

[30] Venkaiah K, Brahmam GN, Vijayaraghavan K. Application of factor analysis to identify dietary patterns and use of factor scores to study their relationship with nutritional status of adult rural populations. J Health Popul Nutr. 2011;29:327–38. 21957671 10.3329/jhpn.v29i4.8448 PMC3190363

[31] Indian Council of Medical Research - National Institute of Nutrition. Dietary Guidelines for Indians. Hyderabad: Indian Council of Medical Research - National Institute of Nutrition; 2024.

[32] Kinra S, Bowen LJ, Lyngdoh T, Prabhakaran D, Reddy KS, Ramakrishnan L, et al. Sociodemographic patterning of non-communicable disease risk factors in rural India: a cross sectional study. BMJ. 2010;341:c4974. 20876148 10.1136/bmj.c4974 PMC2946988

[33] Cribb VL, Jones LR, Rogers IS, Ness AR, Emmett PM. Is maternal education level associated with diet in 10-year-old children? Public Health Nutr. 2011:1–12. 10.1017/S136898001100036X 21414248

[34] Bowen L, Ebrahim S, De SB, Ness A, Kinra S, Bharathi AV, et al. Dietary intake and rural-urban migration in India: a cross-sectional study. PLoS ONE. 2011;6:e14822. 21731604 10.1371/journal.pone.0014822 PMC3120774

[35] Dutta M, Selvamani Y, Singh P, Prashad L. The double burden of malnutrition among adults in India: evidence from the National Family Health Survey-4 (2015-16). Epidemiol Health. 2019;41:e2019050. 31962037 10.4178/epih.e2019050 PMC6976728

[36] GBD 2021 Forecasting Collaborators. Burden of disease scenarios for 204 countries and territories, 2022-2050: a forecasting analysis for the Global Burden of Disease Study 2021. Lancet. 2024;403:2204–56. 38762325 10.1016/S0140-6736(24)00685-8 PMC11121021

